# Advances in seed conservation of wild plant species: a review of recent research

**DOI:** 10.1093/conphys/cot030

**Published:** 2013-11-04

**Authors:** Fiona R. Hay, Robin J. Probert

**Affiliations:** 1 T.T. Chang Genetic Resources Center, International Rice Research Institute, DAPO Box 7777, Metro Manila, Philippines; 2 Seed Conservation Department, Royal Botanic Gardens Kew, Wakehurst Place, Ardingly, West Sussex RH17 6TN, UK

**Keywords:** *Ex situ* conservation, seed bank, seed storage, wild plant species

## Abstract

Seed banking is now widely used for the *ex situ* conservation of wild plant species. Many seed banks that conserve wild species broadly follow international genebank guidelines for seed collection, processing, storage, and management. However, over the last 10–20 years, problems and knowledge gaps have been identified, which have led to more focused seed conservation research on diverse species. For example, there is now greater ecogeographic understanding of seed storage behaviour and of the relative longevity of orthodox seeds, and we are therefore able to predict which species should be conserved using cryostorage techniques; seed development studies have identified when seeds should be harvested for maximal tolerance of desiccation and longevity in storage, as well as highlighting how seed development can vary between species; and there is now a wealth of literature on the dormancy-breaking and germination requirements of wild species which, as well as enabling better management of accessions, will also mean that their use in restoration, species reintroduction, or for evaluation for other applications is possible. Future research may be focused, for example, on nursery and plant production systems for wild plant species that maximize genetic diversity, so that introduced seeds and plant materials have the resilience to cope with future environmental stresses.

## Abstract (precis)

The importance of wild plant diversity for future food security, human health and ecosystem function and repair is generally accepted. Seed banking is widely used to safeguard wild species and research into the complexity of seed behaviour has led to changes in how seeds of wild species are handled in genebanks.

## Introduction

There are more than 1750 seed banks in the world established for the *ex situ* conservation of plant diversity, the majority of which conserve crop diversity, storing a combined total of about 4.6 million accessions of 64 food and forage crops covered by the multilateral system of benefit sharing of the International Treaty on Plant Genetic Resources for Food and Agriculture (FAO, 2010). These seed banks distribute seed germplasm to crop scientists and researchers around the world, and the seed is germinated as the first step in the quest for genes to improve quality, to improve yield, and/or to overcome biotic or abiotic stresses. Most seed banks conserve germplasm of a range of crop species and, perhaps, their closest wild relatives (crop wild relatives or ‘CWR’); relatively few are focused on a single crop. Other seed banks conserve germplasm of other species of global or national economic importance, including that of horticultural crops and fruit and timber trees.

Seed banking has also been used for the *ex situ* conservation of wild species that are not CWR. Since the Global Strategy for Plant Conservation (GSPC) was adopted in 2002, many thousands of samples of seeds from wild species have been placed into long-term storage, in no small part due to the efforts of the Millennium Seed Bank Partnership (Secretariat of the Convention on Biological Diversity, 2009). These collections may be stored in facilities dedicated to the conservation of wild plant species, such as the Millennium Seed Bank (MSB) and other seed banks maintained by botanic gardens and similar institutions, or may be stored alongside national crop collections. One of the revised targets of the GSPC is for at least 75% of globally threatened plant species to be included in *ex situ* collections and at least 20% available for recovery and restoration programmes by 2020.

Seed banks generally store seeds according to the genebank standards (FAO/IPGRI, 1994; FAO, 2013); there are no specific standards for the conservation of seeds from wild plant species, and most of the theory is derived from studies on crops, except for occasional in-depth studies on particular species, perhaps chosen because an accession has not behaved as expected. Whilst most of the protocols are probably acceptable for cultivated and non-cultivated species alike, there are some fundamental differences between the two, which means that methods may need to differ (or indeed may not be required; Table 1). For example, clearly an accession of a wild species that is not a CWR is unlikely to be requested as much as an accession of a crop species; it is therefore not necessary to maintain seed samples from a non-CWR wild species in both an ‘active’ (medium-term storage, in which viability is maintained at ≥65% for 10–20 years; FAO/IPGRI, 1994) and ‘base’ (long-term storage) collection. In contrast, the cost of storing a large volume of material in long-term storage is high (due to the lower storage temperature), and it is more cost effective, for crop accessions, to store the bulk of the material that is going to be distributed within a few years in medium-term storage. Another example where seed bank procedures may differ arises from the fact that, whilst traits that have been favoured for many crop species include shattering resistance and uniformity in flowering and seed maturation, many wild species readily disperse their seeds and have indeterminate flowering. This, and the fact that populations of wild species are unlikely to be found growing in isolation across a large area of land, means that it may be difficult to collect a large quantity of seeds, and what is collected may have variable maturity; this can cause problems during processing and may limit the number of seeds available for storage, testing, and distribution.

**Table 1:** Summary of some of the differences between cultivated and wild species that influence our ability to store, manage, and use accessions of the latter and some of the potential future foci for further research

	Cultivated species	Wild species	Future research areas for wild species accessions
*Ability to store*			
Seed storage behaviour	Majority known to have orthodox seeds. For non-orthodox species, appropriate propagation and conservation methods are available	Majority expected to have orthodox seed storage behaviour, but it may not always be known. Storage behaviour may be predicted based on physical attributes of seeds/fruits; otherwise, desiccation tolerance experiments are needed	Seed storage behaviour continues to be determined for diverse species. Such knowledge is likely to improve predictive models of storage behaviour and phylogeny-/ecology-based understanding of the occurrence of each category of seed storage behaviour
Seed development	Flowering may be simultaneous across a population. Cultivated species often have some degree of resistance to shattering, giving a window of opportunity for seed collection. Seed development studies may have already established optimal time to collect for maximal longevity in storage	For most species, seeds are readily dispersed, and there is a narrow window of opportunity to collect. Indeterminate flowering means that it may be difficult to collect many seeds or that seeds will inevitably be collected at a range of maturities. Indicators of fruit/seed maturity may not be obvious. Pattern of seed development may not be typical (e.g. commencement of seed dispersal before all seeds have acquired desiccation tolerance)	Post-harvest maturation treatments may be applied more routinely for wild species collections. Seed development studies may be necessary for species where problems are identified which are attributed to high proportions of immature seeds
Seed processing and storage	Seeds often regenerated (and hence harvested) close to appropriate processing facilities (e.g. for seed drying). Volume of material collected for each accession means that some operations (e.g. threshing, cleaning) may be automated. Appropriate to store in both medium- and long-term storage conditions	Wild species seed-collecting trips may be long (weeks) and some distance away from appropriate drying facilities, resulting in declines in seed quality. Number of seeds collected may be small, meaning that options for automation are limited. The relatively low number of seeds, low rates of distribution, and unknown longevity in storage mean that long-term storage conditions (or in liquid nitrogen) are most appropriate	Seed-collecting equipment may include a desiccant-based system for drying seeds during transit
*Ability to manage*	Relative longevity during storage may be known and/or genebank data published to help predict longevity. Germination protocols are available. Vigour tests, already widely used for some species, may also prove useful for identifying ageing seed lots. Regeneration guidelines are available and already routine	The relative longevity of seeds of the vast majority of wild species is not known and may vary considerably even within a species due to population differences and/or environmental effects. Some wild species have been found to produce seeds that are extremely short lived in storage (while others are long lived)	Comparative longevity studies will probably continue, resulting in greater understanding of how much seed longevity varies among species and between seed lots of the same species, aiding the effective management of wild species accessions
*Ability to use*	Good understanding of requirements for dormancy breaking, germination, and field establishment	Methods for multiplying material, either for storage or for use, that maintain genetic diversity have not been established and may be species specific. Significant levels of attrition can occur during establishment, causing reduction in yield and genetic diversity. Seeds may not be produced for several years following the planting of original material	Establishing protocols to use wild species accessions for restoration and species reintroduction is an expanding area of research across a number of disciplines, including horticulture and soil science. More focus may also be given to the potential use of conserved species, e.g. as alternative food or industrial crops or for medicinal purposes

It is expected that seed bank collections of wild species will play an increasingly important role in habitat restoration and reintroduction of species (Merritt and Dixon, 2011). Hence, it is vital that collections of wild species are managed effectively and that sufficient viable seeds are available for use (or for producing larger volumes of seeds). This review will consider the current knowledge that is available to guide the management and use of wild species collections in seed banks.

## Ability to store

### Seed storage behaviour

Seed-bearing species may, in most cases, be grouped into one of the following three categories of seed storage behaviour: recalcitrant (desiccation intolerant); intermediate (partly desiccation tolerant and sensitive to low temperature); or orthodox (desiccation tolerant). Non-orthodox seeds cannot be stored successfully long-term using conventional genebank protocols (drying and storage at low temperatures). Rather, cryopreservation (usually in liquid nitrogen, at −196°C) is recommended, and the technology for such storage has advanced sufficiently that it should become a routine activity for the *ex situ* conservation of non-orthodox species (Walters *et al.*, 2013).

Orthodox seeds are those that can be stored in ‘conventional’ seed banks. Such seeds tolerate drying to very low moisture contents (≤3–7% fresh weight), and their longevity increases as moisture content and temperature are reduced (Roberts, 1973). Fortunately, most flowering plant species, including most major and minor food and agricultural crops, do produce seeds that are orthodox. In May 2011, the Seed Information Database (Royal Botanic Gardens Kew, 2008) listed the seed storage behaviour for 19 676 species, of which 93.9% were described as having (or probably having, based on the data available) orthodox seed storage behaviour; relatively few were described as having recalcitrant (2.8%) or intermediate (0.8%) seed storage behaviour (K. Liu, personal communication).

If the seed storage behaviour of a species has not been documented, it may be determined by drying samples of freshly harvested seeds of high (and known) initial viability to low moisture content and storing a sample of those seeds at a temperature below 0°C, before carrying out a germination test (Hong and Ellis, 1996; Pritchard *et al.*, 2004). The procedure for the germination test must overcome any dormancy in the seeds (e.g. Wood *et al.*, 2000) or any other physical effects that may have arisen during drying and/or storage and have a negative impact on germination. For example, dry seeds of some species can be vulnerable to imbibition injury and must be rehydrated gently before exposure to liquid water (Bochicchio *et al.*, 1991), and seeds of *Cuphea* P. Browne spp., which were thought to have intermediate storage behaviour, were able to germinate following low-temperature storage if they were initially exposed to a heat pulse (45°C), which allowed the melting of medium-chain-length saturated fatty acids that crystallize at sub-zero temperatures (Crane *et al.*, 2003).

If it is not possible to carry out the necessary experiments to determine seed storage behaviour, it may be predicted according to taxonomy, origin, and other seed traits. Although some plant families or genera have representatives showing each category of seed storage behaviour, others may comprise species showing only orthodox or recalcitrant behaviour (Royal Botanic Gardens Kew, 2008). Non-pioneer, evergreen rain forest tree species have the highest frequency of recalcitrant seed storage behaviour; the frequency declines as the habitat of origin becomes drier (Tweddle *et al.*, 2003). Recalcitrant seeds are also likely to be dispersed during the wet season, at higher moisture contents than orthodox seeds, and to germinate readily because there is little or no slowing down of metabolism and development of dormancy common in orthodox seeds; they are also likely to be relatively large and have relatively thin outer tissues (Tweddle *et al.*, 2003; Daws *et al.*, 2005; Ellis *et al.*, 2007; Berjak and Pammenter, 2008). Daws *et al.* (2006) developed these ideas further and used data from 104 woody species from a Panamá forest to fit a model to predict seed desiccation sensitivity based on overall seed mass and the relative mass of the seed coat. Seed storage behaviour continues to be determined experimentally for diverse species (e.g. Xia *et al.*, 2012; Han and Sun, 2013; Jayasuriya *et al.*, 2013) and, if these traits are routinely collected in such studies, this model might be refined (or indeed, refuted). For the time being, while it may not be possible to make accurate measurements of these traits in the field, it should nonetheless be possible to identify species that could potentially show non-orthodox seed storage behaviour, for which making a large collection for conventional seed bank storage might be a waste of time and resources, without first carrying out tests to check for desiccation sensitivity.

The subsequent sections of this review focus on orthodox seeds, because it is these seeds which may be stored in conventional seed banks.

### Importance of seed maturity

**Figure 1:**
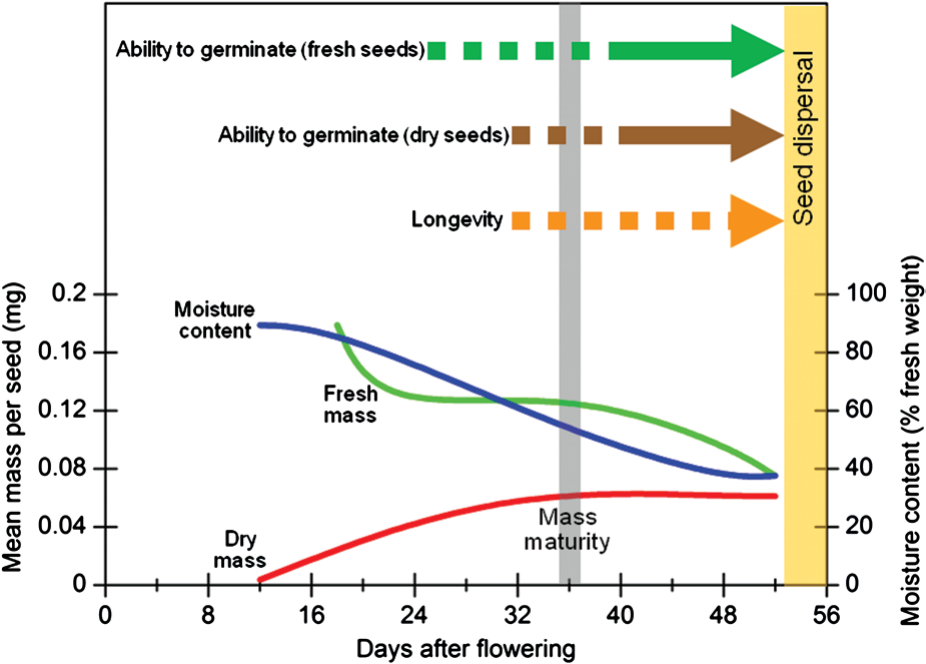
Schematic diagram showing the pattern of seed development for orthodox seeds of foxglove (*Digitalis purpurea* L.) based on original data from Hay (1997). Mass maturity is defined as the point when maximal dry weight is reached (Ellis and Pieta Filho, 1992). The dashed and continuous parts of the arrows indicate the time when the trait (the ability to germinate before or after drying, and longevity) is increasing and stable, respectively.

There have been numerous studies on the development of seeds of crop species to identify the optimal time to harvest for maximal quality (e.g. Demir and Ellis, 1993; Rao and Jackson, 1996; Sanhewe and Ellis, 1996a, b; Sinniah *et al.*, 1998; Eskandari, 2012). In contrast, only a few studies have considered the development of seed quality for non-cultivated species (Hay and Probert, 1995; Hay, 1997; Ali *et al.*, 2007; Newton, 2011). The general pattern of seed development in terms of changes in seed fresh and dry mass, ability to germinate before and after rapid drying, and longevity is shown in Figure 1. Seeds of most orthodox species acquire the ability to withstand drying after they have acquired the ability to germinate when fresh (i.e. without drying), around the time when they acquire maximal dry weight (‘mass maturity’; Ellis and Pieta Filho, 1992), although some cereals have been found to acquire desiccation tolerance earlier (reviewed by Hay and Smith, 2003). The cessation of the accumulation of dry weight is caused by the termination of the vascular connection between the seed and the maternal plant; the amount of water in the seed also starts to decline after this time, as the seed starts to equilibrate to the ambient conditions. During this desiccation phase, seed quality, in particular with respect to longevity in air-dry storage, continues to increase (Figure 1). In the case of non-cultivated species with dry, dehiscent fruits, the seeds are likely to be dispersed at the point when equilibrium with the microclimate is reached, and it is recommended that this would be the optimal time to harvest for maximal subsequent longevity in seed bank storage (Hay and Probert, 1995; Hay and Smith, 2003). Determining the equilibrium relative humidity of a sample of seeds will confirm whether or not seeds have equilibrated with ambient conditions (Hay and Probert, 2011). As well as looking for signs that seed dispersal has or is about to commence within the target population, there may be other indicators of relative maturity, including changes in fruit or seed colour (Hay and Smith, 2003; Hay *et al.*, 2010; Vidigal *et al.*, 2011; Newton *et al.*, 2013).

Unfortunately, unless the species being collected is serotinous or otherwise resistant to shattering, the fact that seeds are likely to be readily dispersed means that they are often collected before they are fully ripe. Furthermore, given the variability in flowering time for many wild species, both within and among individual plants in a population, it is inevitable that a seed collection will contain a significant proportion of seeds that are less mature. The most immature seeds might not survive the enforced, post-harvest seed drying (Figure 1) and, while it may be possible to remove under-sized (potentially desiccation-intolerant) seeds during seed cleaning, this is not ideal if it is difficult to collect a large number of seeds, and the overall quality of the seed lot will be compromised. For such seed lots, which clearly contain significant levels of immature seeds, a post-harvest ripening treatment can be applied, using conditions of high humidity (≥75% relative humidity) and a temperature regimen similar to that which the seeds would experience *in situ* (Hay and Probert, 2011; FAO, 2013). Seeds of some species may even need to be kept fully hydrated post-harvest to allow maturation to proceed. Seeds of *Anemone nemorosa* L. have been found to have an unusual pattern of development in that, at the commencement of natural dispersal, a high proportion of seeds have yet to acquire desiccation tolerance (Ali *et al.*, 2007). Seeds buried in the leaf litter at the collection site or placed on 1% water agar at 20°C in the laboratory continue to acquire but then gradually lose desiccation tolerance as the under-developed embryos grew within the seeds. Likewise, only a proportion of the seeds of *Narcissus pseudonarcissus* L. seed lots collected at the point of natural dispersal were able to tolerate seed bank drying conditions (Newton *et al.*, 2013). Post-dispersal acquisition of desiccation tolerance may occur in other species that occur in cool, damp habitats and whose seeds have under-developed embryos at the time of seed dispersal. Clearly, this is an area that needs further study if accessions with high viability and good storage potential are to be placed into long-term seed bank storage.

### Seed processing and storage

The procedures recommended for processing seeds prior to banking, i.e. drying, cleaning/sorting, and seed health and viability testing, are well documented (FAO/IPGRI, 1994; Rao *et al.*, 2006; FAO, 2013). However, cleaning and sorting seeds, and other activities, may still be predominantly manual operations in many seed banks. Part of the reason for this is that seed banks are often processing relatively small quantities of each accession, and accessions may be highly diverse, reducing opportunities for automation. This is particularly true of wild species seed banks, where seed size and morphology are likely to vary considerably from one accession to the next. Furthermore, seed banks, particularly those with a mandate to conserve germplasm as a global public good, are expected to adopt the international standards for genebanks (FAO/IPGRI, 1994; FAO, 2013) and perhaps do not have the resources to evaluate whether they truly are optimum for maintaining and managing collections or may do so only when problems arise.

To some extent, commercial seed companies and seed bank managers have similar goals; that is, to maximize the physical purity, health, and physiological quality of their seeds. For the commercial seed companies, this is because high-quality seeds (seeds with high, fast, and uniform germination) have greater monetary value, whereas for seed bank managers the purpose is to maintain maximal genetic diversity, and the potential value of an accession is unknown. Increasingly sophisticated seed-sorting equipment is being developed, involving sorting of individual seeds based on image analysis of external traits (size, aspect ratio, colour, etc.), internal morphology (subjecting seeds to X-rays), and/or composition (Dell'Aquila, 2009; Deleuran *et al.*, 2013). However, with any automated sorted technology, the risk of introducing genetic drift must be evaluated. For example, would sorting based on seed size result in the loss of alleles related to seed size? Some seed-sorting machines may already be suitable for seed bank accessions, at least for pre-sorting (with final sorting through visual inspection by trained personnel), or it may be possible for them to be adapted and/or scaled down to meet the needs of a seed bank handling smaller seed bulks.

The standard for seed drying is derived from the expectation that seed longevity during storage will be maximized when the seeds have been dried to a moisture content that is in equilibrium with 5–20°C and 10–25% relative humidity (FAO, 2013). Hence, most seed banks will typically place incoming seeds into a chamber or drying room set within these limits of relative humidity and temperature. As discussed in the previous section, immature seeds may be better placed in conditions that simulate those that the seeds would experience *in situ* before final equilibration prior to packing for long-term storage. Desiccants such as silica gel, calcium chloride, charcoal, and zeolite beads may also be used for drying seeds to low moisture contents (Probert, 2003; Rao *et al.*, 2006; Hay *et al.*, 2012) and may be particularly useful for drying seeds in the field, during a collecting trip, by placing the seeds in net or cloth bags in a sealed container with desiccant. If the weather conditions are warm and dry (<40% relative humidity; Rao *et al.*, 2006) during a collecting trip, an effective amount of drying may be possible by spreading seeds in a monolayer on linen or mesh sheets and placing them in the shade. Drying without shade is possible, but care must be taken to avoid over-heating, which might cause, for example, cracking (Probert, 2003).

Requests for seed bank samples of wild species, especially of non-CWR species, are likely to be less frequent than those for crop and CWR species. For example, the T.T. Chang Genetic Resources Center at the International Rice Research Institute (IRRI) distributed more than 10 000 accessions of cultivated rice to users outside of IRRI in 2012, compared with less than 1000 accessions of wild rice species. In the same year, the MSB distributed only 868 samples. Such figures emphasize why it is useful to have samples of crop accessions in both medium-term storage (the active collection, with the bulk sample of seeds that will be used for distribution) and long-term storage (the base collection, holding a smaller quantity of seeds of each accession), with all accessions present in both, while long-term storage conditions alone, as used by the MSB, might be sufficient and most appropriate for wild species. Crop genebanks are likely to store much smaller quantities of CWR accessions than they do of cultivated accessions. This may be due to lower demand but also because it can be difficult to grow wild species, and seed production rates may be very low. With increase in demand for samples of wild species, it may be necessary to have larger quantities available. Merritt and Dixon (2011) suggested that ‘restoration seed banks’ might need to be able to supply tens to hundreds of tons of seeds for restoration projects. These sorts of quantities are unlikely to be placed into long-term seed bank storage conditions; if multiplication is demand driven, short-term storage following multiplication, in conditions that will maintain viability at adequate levels for a few years, may suffice.

**Figure 2:**
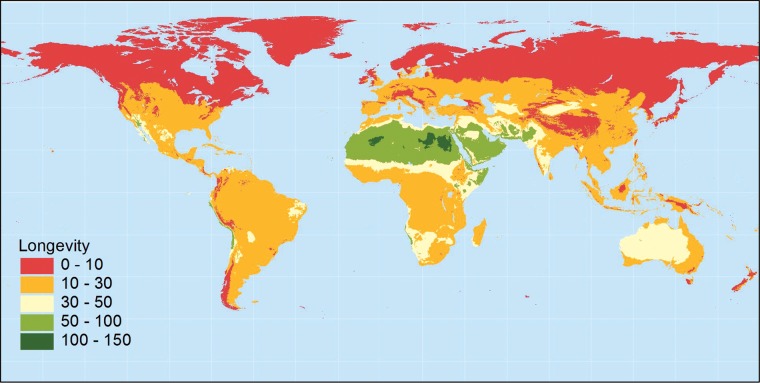
Map of the world showing the predicted relative longevity of endospermic seeds depending on the climate at the origin of the seed lot and based on the relationships published by Probert *et al.* (2009). Predictions of longevity are the estimated time for viability to fall to 50% (*p*_50_, in days) during storage at 60% relative humidity and 45°C. The general pattern would be the same for non-endospermic seeds, although their longevity is expected to be greater. Relative seed longevity (ranking of species) is expected to be similar in seed bank storage conditions, i.e. a seed lot with short-lived seeds in experimental storage conditions is expected to be relatively short lived in seed bank storage conditions. This map was created in May 2013 by A. Nelson (International Rice Research Institute) using WorldClim global climate data (http://worldclim.org/).

Another reason why it makes sense to place wild species accessions into long-term storage conditions alone is that the longevity of the seeds in storage is likely to be unknown. The rate of decline in the viability of seeds has been statistically modelled using the Ellis and Roberts (1980a) viability equations for fewer than 100 species, most of which are crops (Royal Botanic Gardens Kew, 2008), and most publications containing genebank retest data (the results of germination tests carried out to monitor viability) have likewise focused on accessions of cultivated material (e.g. Walters *et al.*, 2005; Niedzielski *et al.*, 2009; van Treuren *et al.*, 2012; Hay *et al.*, 2013). Storage experiments, placing samples of seeds in conditions of relatively high moisture and temperature, and monitoring their viability, have shown that relative seed longevity can vary enormously across diverse taxa (Probert *et al.*, 2009). Seeds from species within certain plant families or genera appear to be typically short or long lived, and seeds from species originating in cool, wet environments are likely to have shorter lifespans than those from warm, dry environments (Figure 2; Probert *et al.*, 2009). Mondoni *et al.* (2011) further demonstrated the short longevity of seeds from alpine populations compared with those from lower-altitude populations of the same or closely related taxa. Seed lots of species with the shortest-lived seeds may survive only a year or two at best, even in conventional long-term storage conditions (Ali *et al.*, 2007). Cryopreservation may be the only recourse to ensure the effective *ex situ* seed conservation of such species (Li and Pritchard, 2009; FAO, 2013).

## Ability to manage

Monitoring the viability of seeds during storage, by removal of a sample for germination testing, is an essential aspect of effective management of seed bank collections. While the 1994 genebank standards recommended monitoring viability every 5 or 10 years for seeds in medium- or long-term storage, respectively (FAO/IPGRI, 1994), the revised standards recommend that, if deterioration periods can be predicted [e.g. using the Ellis and Roberts (1980a) viability equation], viability monitoring should be carried out at intervals one-third of the time predicted for viability to fall to the regeneration standard (FAO, 2013). Incorporating variable retest intervals into seed bank management software is, of course, possible; the difficulty is in deciding on the method behind setting retest intervals, particularly given that the parameters of the Ellis and Roberts (1980a) viability equations have been determined for only a small number of wild plant species. Furthermore, there have been some cases where at least one of the so-called ‘species constants’ of the viability equations have (or have by inference) differed between different seed lots of a species, due to differences in maturity at harvest or growing environment (Hay *et al.*, 1997; Kochanek *et al.*, 2010; Mondoni *et al.*, 2011). In addition, even if species constants are known and stable, the longevity of a particular seed lot will depend on the initial quality of the seeds when first placed into storage (Probert *et al.*, 2007).

Crawford *et al.* (2007) summarized seed bank retest data for 124 accessions of 72 native Australian species, of which, after 5–12 years stored at −20°C, only 12 accessions from 10 species showed a significant decline. They speculated that the relatively rapid loss of viability apparent for a few seed lots was accession specific, because in some cases, other accessions of the same species did not show a significant decline. Likewise, Godefroid *et al.* (2010) found that loss of viability during storage in the seed bank of the National Botanic Garden of Belgium could be seed lot specific. Thus, it seems that for the management of wild species accessions, caution should be urged and regular monitoring carried out until such time that there is assurance that viability is being maintained or until there are sufficient data to predict when viability will reach the critical level when regeneration should be performed. The alternative option, to test the relative longevity of a sample of seeds at the start of seed bank storage by conducting a storage experiment using a standard comparative longevity protocol (45°C, 60% relative humidity; Newton *et al.*, 2009), is unlikely to be practical, particularly for accessions with a small number of seeds, because it is destructive. A streamlined version of the comparative longevity protocol, designed to screen for short-lived species, has been developed at the MSB. This test uses 200 seeds (rather than 500), and seed viability is monitored at only four intervals during controlled ageing. Estimates of the length of time for viability to fall to 50% did not differ significantly depending on the version of the comparative longevity test used for 21 of 27 seed lots (wild species accessions, selected at random; R.J. Probert *et al.*, unpublished data). Significantly different estimates of longevity were obtained for one of the other seed lots; three did not show a significant decline in germination in either test; and for two seed lots, there were too few data to determine longevity parameters; however, both methods would have identified the seed lot to have been short lived. Plans are now in place to introduce the streamlined test for routine screening of putative short-lived species collected for long-term conservation at the MSB.

Efficient viability monitoring through a germination test may still be hampered by a lack of knowledge of dormancy-breaking and germination requirements (see following section). Godefroid *et al.* (2010) suggested that viability testing, for example by doing a ‘cut test’ at the end of a germination test to see whether non-germinated seeds are still fresh and healthy (and hence probably dormant), empty, infested, or soft and mouldy (i.e. dead) should be used to evaluate the quality of accessions. However, one of the problems associated with germination tests on wild plant species is that incubation periods can extend to many weeks or months. Consequently, there is a risk that some seeds might die during the test, especially if germination conditions are not optimal. One way around this problem is to perform cut tests on seeds that have been imbibed for a few days. Alternatively, a tetrazolium test, in which viable seed tissues become stained a dark red, may also be used to verify viability where germination results are poor (ISTA, 2013). Tetrazolium testing is routinely used at the MSB (Terry *et al.*, 2003).

Seed vigour tests are very important to the seed industry; seed traders need to know the quality of the product (the seed lots) being exchanged. The vigour of a seed lot is a trait which encompasses the likelihood and rate at which a seed will germinate and whether the resulting seedling will develop into a healthy plant. Seed vigour tests essentially measure the extent of seed ageing that has occurred, although their precise interpretation in terms of how they translate into results in the field may vary among species, varieties, or variety groups (including production methodology, e.g. open pollinated, self pollinated, or hybrid). Traditional measures of seed vigour are calculated from the results of a seed germination test and essentially measure the speed of germination, because the speed will slow down as seeds age. These germination parameters include single counts of the proportion of seeds that have germinated after a set period of time in the germination test and expressed as mean germination time (MGT; Ellis and Roberts, 1980b) or germination index (GI; Maguire, 1962). Precise calculation of these parameters requires accurate and regular observation, which has led to the development of automated systems to follow the progress of germination in a sample of seeds based on image capture and analysis (Matthews *et al.*, 2012). In a seed bank context, especially for crop genebanks carrying out thousands of tests a year, such an automated system could both improve the accuracy of the percentage germination result (avoiding miscounts at the scoring and/or sowing stages) and save a lot of person hours. Furthermore, while most seed banks normally record only the percentage germination result, an automated system would enable the accurate and fast determination of a measure of the speed of germination; this could be used as an indicator of seed ageing before loss of viability is apparent (Powell and Matthews, 2012).

Over recent years, advances have been made in understanding the seed ageing process, i.e. the reactions that take place within the seeds that lead to declines in vigour and eventual loss of ability to germinate. Much of the damage that accumulates in seeds during storage is attributed to oxidation by reactive oxygen species (Hendry, 1993; Bailly, 2004; Kranner *et al.*, 2010). Ageing processes are slowed in seed bank storage conditions, because the seeds enter a glassy state, and variation in seed longevity between species (or seed lots) may be due to the properties of that glassy state (Walters *et al.*, 2010), efficiency of anti-oxidant systems, and/or the ability to repair damage at germination (Nandi *et al.*, 1997; Kibinza *et al.*, 2006; Waterworth *et al.*, 2010; Chen *et al.*, 2012; Châtelain *et al.*, 2013; Donà *et al.*, 2013). This area of research may lead to a biochemical marker of viability loss that could be used as an alternative to more time-consuming germination tests. Like a germination test, however, such a marker may still be destructive. Alternatively, it has been found that seeds can produce volatile compounds during storage (e.g. Zhang *et al.*, 1993; Mira *et al.*, 2010; Colville *et al.*, 2012), and this may form the basis of a non-destructive test for viability prediction (Mira *et al.*, 2010; Colville *et al.*, 2012).

Crop seed banks are well practiced in the multiplication and regeneration of accessions according to established guidelines (FAO/IPGRI, 1994; Rao *et al.*, 2006; FAO, 2013). In contrast, regeneration of non-CWR wild species by seed banks has probably been sporadic at best and targeted at species which are either more immediately facing extinction in the wild and/or which produce very few seeds. Issues regarding our ability to use wild species accessions (see following section) also apply to our ability to regenerate material for seed bank storage. Perhaps of particular concern, if a species produces seeds that are short lived in storage, is how many cycles of regeneration are acceptable and how to ensure that allelic variation is maintained such that viable living populations could be established if needed in the future.

## Ability to use

One of the major impediments to the potential use of wild species germplasm for species reintroduction or habitat restoration (as well as causing difficulties for viability testing) is lack of knowledge of how to break dormancy and germinate the seed. There is, however, a huge body of literature that can be searched for guidance, if not for the species of interest, then for closely related taxa. Some of this literature has already been compiled (Baskin and Baskin, 2001; Royal Botanic Gardens Kew, 2008), and seed banks may also publish their own germination data (e.g. Wood, 2012). Alternatively, if information is not available and a seed lot is found to be dormant (fail to germinate with a month), it is recommended that seeds are put into a ‘move-along’ experiment that simulates the natural habitat (Baskin and Baskin, 2001, 2003). This approach has been successful in overcoming dormancy for a wide range of species (e.g. Karlsson *et al.*, 2005; Albrecht and McCarthy, 2006; Newton, 2011; Mattana *et al.*, 2012). While Baskin and Baskin (2001) advise using fresh seeds (seeds that have not been dried), this move-along approach has also been effectively incorporated into the routine testing of new accessions at the MSB (accessions that have already been dried, cleaned, and stored at −20°C for at least 1 month).

There have been numerous reports linking germination requirements and seed dormancy to local climate. For example, Mott (1972) found that the optimal temperatures for germination varied in species adapted to grow during either winter or summer rains in Western Australia. Germination and emergence phenology is also finely tuned to local climate in the temperate woodland geophyte *Anemone nemorosa*. In this species, embryo development and germination occurred earlier and at lower temperatures in seeds from a mountain population compared with seeds from lowland populations (Mondoni *et al.*, 2008). In some cases, germination linked to habitat appears to be under genetic control (Meyer and Kitchen, 1994; Meyer *et al.*, 1995, 1997); however, there is also good evidence that germination requirements are strongly influenced by maternal environment (Andersson and Milberg, 1998; Hoyle *et al.*, 2008).

Knowledge of the local climate can therefore be a valuable tool in the prediction of seed germination requirements for wild plant species and, now that climate data for geo-referenced locations are readily accessible from a number of online sources, such as WorldClim (http://worldclim.org/), predictive models are being developed. For example, monthly temperatures and rainfall patterns for the location of conservation collections are being used successfully by the Millennium Seed Bank Partnership to predict optimal temperatures for germination and the duration of dormancy-breaking treatments for seeds likely to possess physiological dormancy (http://www.kew.org/science-research-data/databases-publications/uk-germination-tool-box/).

Dormancy is likely to be lost during storage, and the conditions required for germination (in particular, temperature) become less specific (Probert, 2000), although the rate of loss of dormancy is likely to be slower in seed bank storage than it would be in ambient conditions (Roberts, 1988). As well as loss of dormancy during seed bank storage, induction of dormancy can also occur (e.g. Pérez-García *et al.*, 2007, 2009), and there have been instances where accessions stored in the MSB have failed in a germination retest carried out using the same treatments and/or conditions that were found to be optimum at the start of storage.

Even when a reliable protocol is available for dormancy breaking and germination in controlled conditions, it may still be problematic to regenerate sufficient ‘ready-to-go’ material (non-dormant seeds or seedlings) for transplantation into the wild or, as highlighted by Merritt and Dixon (2011), to a restoration site. Technologies that are already routine, in particular in the horticultural industry, may improve success rates. For example, seed priming is often used as an invigoration treatment to ensure rapid establishment (Parera and Cantliffe, 1994) and could help to ensure that seeds that are sown *in situ* are able to germinate and establish, particularly if the seeds have already aged during storage (Powell *et al.*, 2000; Butler *et al.*, 2009). Mondoni *et al.* (2013) described a percussion (impaction) treatment to overcome hardseededness of bulk lots of legume seeds for producing seedlings for restoration. This aspect of conservation science, understanding how to produce seedlings from wild species seed bank accessions in quantities sufficient to create viable populations with high genetic diversity, will no doubt expand in the coming years, not least if seed banks are to play a role in restoration and species reintroduction.

Increasing focus may also be directed to how accessions are evaluated for potential use beyond restoration and species reintroduction. The Millennium Seed Bank Partnership has often targeted ‘useful’ wild species that are already being used by local people for construction, medicinal purposes, and food; seed banks will inevitably play a role in expanding the sustainable use of such useful native species.

## Conclusion

The genebank standards (FAO, 2013) give indications of where it may be necessary to relax the standards when handling accessions of wild species, for example regarding sample size (number of seeds stored or tested for viability), storing seeds from different maternal plants separately, having shorter retest intervals (3 years) and/or cryostorage for seed lots that are expected to be very short lived, and regenerating accessions in a similar environment to that at the original source of the collection. Nonetheless, seed banks that are routinely storing seeds of wild species have, by necessity, devised protocols that are effective and practical (e.g. Manger *et al.*, 2003; Probert *et al.*, 2003; Terry *et al.*, 2003), and have had to target research to those areas where knowledge was lacking (e.g. Ali *et al.*, 2007; Pérez-García *et al.*, 2009; Probert *et al.*, 2009; Smith *et al.*, 2011). Seed conservation research will no doubt continue on a variety of topics (Table 1) as seeds of more wild species are collected and stored in seed banks, and as more issues come to light, which is unavoidable given the diversity being considered.
